# The Effects of Magnesium Sulfate on Postoperative Pain in Patients Undergoing Lumbar Spinal Surgery: A Systematic Review of Randomized Controlled Trials

**DOI:** 10.7759/cureus.77352

**Published:** 2025-01-12

**Authors:** Ahmed Almajed, Sami Aleissa, Abdullah Al Harbi, Bdour E AlQahtani, Abdullah Alshehri, Ali A Alhandi, Abdulrahaman Alhadlaq, Majed Abaalkhail, Fahad Alhelal

**Affiliations:** 1 College of Medicine, King Saud Bin Abdulaziz University for Health Sciences, Riyadh, SAU; 2 Orthopedic Surgery, Ministry of National Guard - Health Affairs, King Abdulaziz Medical City, King Abdullah International Medical Research Center, King Saud Bin Abdulaziz University for Health Sciences, Riyadh, SAU; 3 Anesthesia, King Saud Bin Abdulaziz University for Health Sciences, Riyadh, SAU; 4 Orthopedics, King Saud Bin Abdulaziz University for Health Sciences, Riyadh, SAU; 5 Medicine, Ministry of National Guard - Health Affairs, King Abdulaziz Medical City, Riyadh, SAU; 6 Orthopedics, Ministry of National Guard - Health Affairs, King Abdulaziz Medical City, Riyadh, SAU; 7 Orthopedic Surgery, King Abdulaziz Medical City, Riyadh, SAU; 8 Orthopedic Surgery, King Abdulaziz Medical City Riyadh, Riyadh, SAU

**Keywords:** lumbar spine surgery, magnesium sulphate, opioid use, postoperative blood pressure, postoperative pain, vas for pain

## Abstract

Magnesium sulfate is commonly used intraoperatively to reduce postoperative pain in spinal surgery patients, but its effects when administered postoperatively are not well established. This study specifically aimed to assess the effect of magnesium sulfate on a postoperative basis on the incidence of postoperative pain in lumbar spinal surgery patients. This systematic review focuses on assessing the practicality of postoperative magnesium sulfate in the mitigation of pain after lumbar surgery. A comprehensive search was conducted across multiple databases, focusing on studies published between January 2000 and December 2023. The findings emphasize the significant role of magnesium in postoperative analgesia for adult patients undergoing lumbar spinal surgeries, with various administration routes explored, such as infusion, intravenous, or intramuscular. All in all, magnesium sulfate shows potential as an analgesic additive in lumbar surgery, aiding in postoperative pain reduction and decreased opioid use. However, its efficacy assessment is hindered by external variables. Standardized research is crucial to establish its optimal clinical applications and address knowledge gaps.

## Introduction and background

Optimizing surgical results, speeding up recovery, and avoiding complications all depend on effective postoperative pain management. Opioids are still essential for managing pain, but their immediate adverse effects (such as nausea and respiratory depression) and long-term problems (including reliance and tolerance) call for different strategies. An efficient adjuvant for lowering postoperative pain and decreasing the usage of opioids is magnesium sulfate [1-3].

Magnesium sulfate has been a beneficial supplementary element in postoperative anesthesia, by potentiating the analgesic profile postoperatively, attenuating postoperative hyperalgesia, alleviating nausea and vomiting, and reducing the need for perioperative pain relievers [4,5]. Magnesium sulfate is a non-competitive N-methyl-D-aspartate (NMDA) receptor antagonist, which suppresses somatic and autonomic reflex responses elicited by painful stimuli, thereby producing a considerable analgesic impact [6,7]. Over the years, magnesium sulfate has gathered considerable attention among physicians as an adjuvant to other cardinal analgesics by generating promising clinical effects.

Magnesium sulfate has grown to become a staple in many clinical anesthetic practices. Whether that be systemic, topical, intrathecal, or epidural, adjuvant anesthesia has been in frequent use in perioperative situations and pain syndromes alike due to its efficacious qualities. A 2008 study noted a decrease in analgesia requirement post arthroscopic knee surgery in patients receiving a bupivacaine-magnesium solution compared to patients who received either agent as a sole analgesic [8]. Furthermore, a 2009 study recorded a significant reduction in patient-controlled analgesia (PCA) morphine consumption and an increase in analgesia satisfaction rates, as opposed to morphine, in trauma-related lower limb orthopedic surgery patients of the magnesium adjuvant groups [9]. Although established with extremely promising effects, spinal diseases and spinal surgeries present a different set of nuances regarding acute pain and pain control.

In 2017, a substantial 5.2 million spinal surgeries were reported, with trends currently exhibiting a robust increase in spinal surgeries since then [10]. Typical spinal surgery frequently involves extensive tissue manipulation and dissection, which frequently contributes to postoperative pain of significant magnitudes and considerable duration. Notably, postoperative spinal surgery pain has been shown to be drastic and severe for up to three days postsurgery [11,12]. Thus, providing sufficient postoperative analgesia in these patients is crucial in the postoperative period of care among these patients. The precedent pain of spinal diseases, coupled with prolonged use of analgesics and/or opioids, modifies these patients’ degree of pain perception, often exacerbating painful stimuli, which contributes to the intricacies and difficulty of pain management [13,14]. Over the past 20 years, numerous studies analyzing the effects of postoperative magnesium sulfate have been published. However, ambiguity remains still regarding postoperative analgesic consumption, pain intensity, and postsurgical effects and events on patients who underwent lumbar spine surgery. The purpose of this review is to exhibit what is known as of yet and shine a light on areas that require further expounding. Furthermore, the analgesic effect of magnesium sulfate as a postoperative pain management treatment after lumbar spinal surgery needs to be analyzed.

This systematic review aimed to evaluate the current evidence from randomized controlled trials (RCTs) related to the effectiveness and value of postoperative magnesium sulfate as adjuvant analgesics in lumbar spine surgery patients. A comprehensive understanding of the current level of evidence in the literature would help clarify the clinical utility of postoperative magnesium sulfate in lumbar spine surgery and inspire future research.

## Review

Methodology

This study was designed as a systematic review and conducted in line with the protocols specified in the Preferred Reporting Items for Systematic Reviews and Meta-Analyses (PRISMA) [15]. Since this is a review of already published randomized control trials and does not include the processing of individual patient data, institutional review board (IRB) permission is not required. We registered this systematic review prospectively in the International Prospective Registry of Systematic Reviews database (registration number: CRD42024553308).

Study Design

A systematic review of randomized controlled trials was conducted following the guidelines outlined by the Preferred Reporting Items for Systematic Reviews and Meta-Analyses (PRISMA), using the PICO framework, the authors structured their research as follows: population (P) - patients who had lumbar spinal surgery; intervention (I) - postoperative magnesium sulfate supplementation via intravenous, epidural, intra-articular, or peri-articular injection; comparison (C) - placebo or drug other than magnesium sulfate; and outcome (O) - postoperative heart rate and blood pressure, analgesic time, magnesium sulfate and opioid consumption, visual analog scale (VAS), and adverse events [15].

Search Strategy

Three authors (Alharbi, Almajed, and AlQahtani) individually searched for qualified RCTs from the following databases: PubMed, Embase, and Cochrane Library. Additionally, Google Scholar was searched to identify any potentially relevant studies. The search covered the period from January 1, 2000, to December 31, 2023, without language restrictions and was conducted during June 2024. The following terms were searched in the PubMed database: ("magnesium"[MeSH Terms] OR "magnesium"[All Fields]) OR ("magnesium sulfate"[MeSH Terms] OR "magnesium sulfate"[All Fields]) AND ("postoperative"[MeSH Terms] OR "postoperative"[All Fields]) AND ("spine surgery"[All Fields] OR "spinal surgery"[All Fields] OR “back surgery”[All Fields] OR "spine operation"[All Fields] OR "spinal operation"[All Fields] OR "spine fusion"[All Fields] OR “lumbar fusion"[All Fields] OR laminectomy[All Fields] OR Foraminotomy OR Vertebroplasty OR Kyphoplasty OR Microdiscectomy OR discectomy[All Fields]OR "Artificial disc replacement"[All Fields] OR "Spinal decompression surgery"[All Fields] OR "Spine/surgery"[MeSH])ِ AND ("pain"[All Fields] OR "Pain"[MeSH]) NOT ("preoperative"[All Fields] OR "preoperative"[MeSH] AND "perioperative"[All Fields] OR "perioperative"[MeSH] AND "intraoperative"[All Fields] OR "Intraoperative"[MeSH]). The search key terms used in Embase, Cochrane Library, and Google Scholar are summarized in Table 1. To find other relevant publications, a manual search of the bibliographies of all selected articles was conducted throughout the full-text review, as well as forward and backward citation tracking.

**Table 1:** Keywords used for search strategy.

Category	Keywords and MeSH terms
Magnesium sulfate	“Magnesium,” “Magnesium sulfate”
Postoperative	“Postoperative”
Spine surgery	"Spine surgery," "Back surgery," "Spine operation," "Spine fusion," "Lumbar fusion," "Laminectomy," "Foraminotomy," "Vertebroplasty," "Kyphoplasty," "Microdiscectomy," "Discectomy," "Artificial disc replacement," "Spinal decompression surgery," "Spine/surgery"
Pain	"Pain"

Eligibility Criteria

This study included all published randomized clinical trials and prospective or retrospective observational studies that administered magnesium sulfate during the postoperative period for the management of lumbar spinal surgery postoperative pain. No language restrictions were applied. The assessment focused solely on the postoperative administration of magnesium sulfate. Therefore, any studies involving preoperative or intraoperative administration of magnesium sulfate were excluded. The delivery of magnesium by intravenous (IV), epidural, intra-articular, and peri-articular injection (PAI) routes were taken into consideration. Also, any study that assessed oral magnesium supplementation in the control group was excluded. Furthermore, studies that did not evaluate the primary outcomes or those that compared magnesium against different experimental treatments (rather than a control group) were also excluded. Descriptive studies, case-control studies, field studies, animal research, duplicate publications, pilot studies, letters to the editor, individual case reports, case series, longitudinal studies, and review articles were also excluded.

Study Selection

Two authors (Almajed and AlQahtani) independently inspected the titles, abstracts, or full texts of the articles to determine their eligibility, and any duplicates or irrelevant studies were excluded. A third author (Alharbi) resolved any conflict between the two authors. Afterward, the complete texts of potentially suitable studies were acquired, and final decisions were made according to the predetermined eligibility criteria. Any differences in opinion or conflicts were resolved through discussion.

Outcomes of Interest and Definitions

The main outcomes of interest of the present study were postoperative visual analog scale (VAS), patient stratification, magnesium sulfate consumption, opioid consumption, and adverse events during the 24 hours postoperative (PO) period. Postoperative visual analog score refers to the level of pain experienced by the patient in a score of 0-10, where 0 = no pain, 1-3 = mild pain, 4-6 = moderate pain, and 7-10 = intolerable pain. Patient satisfaction is how content a patient is to the analgesic provided. Magnesium sulfate consumption refers to the amount of magnesium sulfate taken by the patient in the case group. Also, the term opioid consumption is used to indicate the quantity of opioid painkillers taken by patients. Adverse events include nausea, vomiting, allergic reaction, dry mouth, respiratory depression, muscular weakness, and urinary retention.

Data Extraction

Three authors (Alharbi, Almajed, and AlQahtani) independently reviewed and extracted the following information from each study into a data extraction form: names and roles of authors, country of origin, publication date, patient data (American Society of Anesthesiologists physical status classification, age, sex), sample sizes in treatment and control groups, surgery type, types of anesthesia, route and dosage of magnesium sulfate administration, cumulative analgesic consumption, visual analog scale score, time to first analgesic request, and adverse events. If additional data are mentioned in the figures, contact with the authors was made to obtain the unwritten data. The data from the figures have been extracted if there is no response from the authors. Once the extraction process was done, a senior researcher (Alshehri) did a double analysis process. 

Risk of Bias Assessment

Two authors (Almajed and AlQahtani) independently evaluated and cross-checked each trial according to the Cochrane Risk of Bias Assessment Scale [16]. Among the researchers, any disparities in grading were discussed. The following five categories of bias were assessed: the randomization process, deviations from planned interventions, missing outcome data, outcome measurement, and the selection of reported results. Three risks of bias levels: low risk (no risk of bias in any category), mild risk (some risk of bias in any category), and high risk (more than one risk of bias in any category).

Certainty of Evidence

The authors used the Grading of Recommendations Assessment, Development, and Evaluation (GRADE) system to evaluate the level of certainty [17]. Each outcome's evaluation scores were then combined and classified as high, moderate, low, or extremely low.

Data Synthesis and Analysis

The data were first collected as previously stated. The analysis process was done with the use of peer-review, Excel (Redmond, WA: Microsoft Corp.), and finally through SPSS (Chicago, IL: IBM Corp.). We defined our inclusivity as follows: (1) lumbar spine surgeries, (2) magnesium sulfate administration, (3) postoperative administration, (4) pain scores, and (5) analgesics use. Most of the 397 studies collected were those that began administration perioperatively, which were not inclusive for the purpose of the present study. Other reasons for removal would include duplicates and surgical sites, other than lumbar spine. 

Results

**Figure 1:**
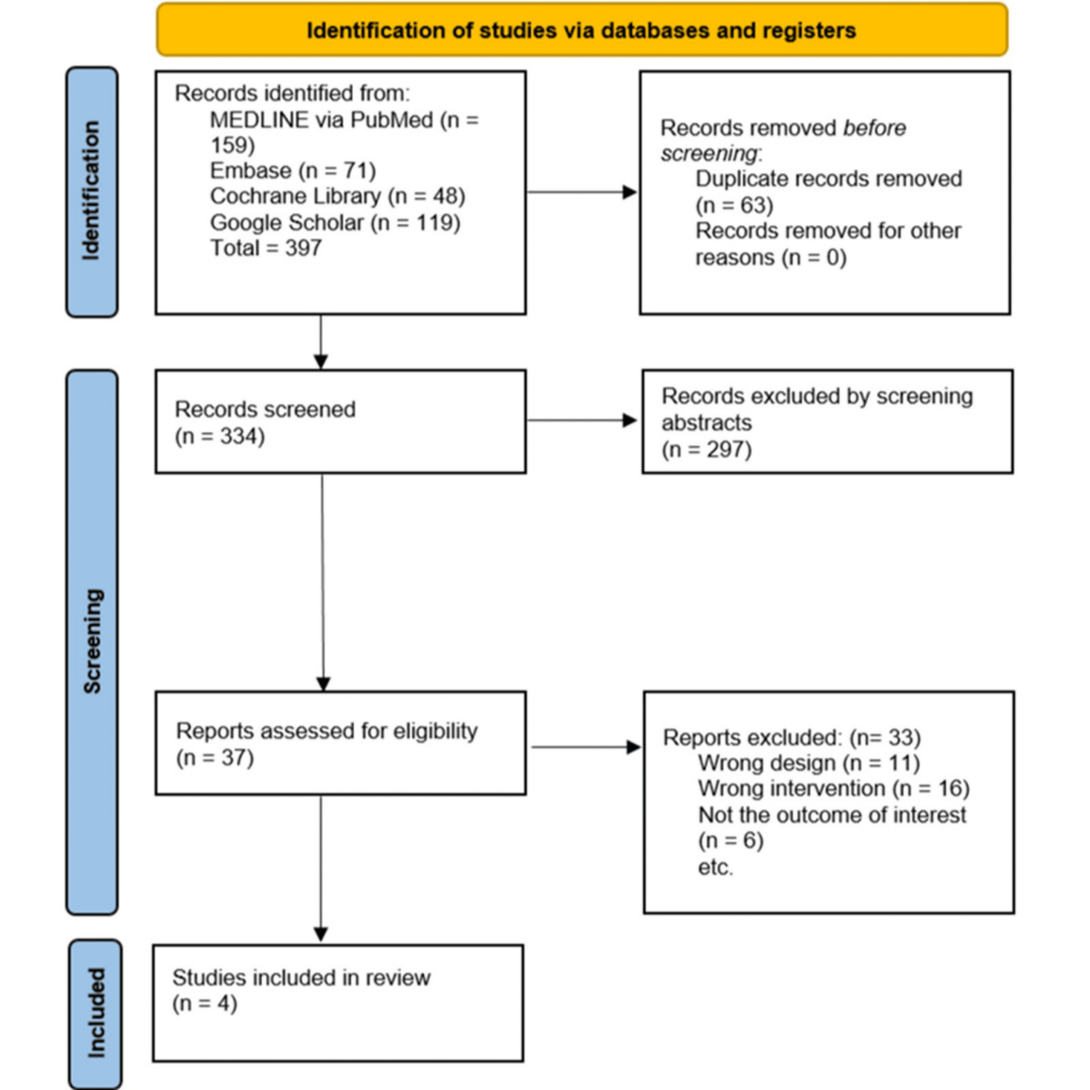
PRISMA flow chart of the study selection process. PRISMA: Preferred Reporting Items for Systematic Reviews and Meta-Analyses

Following Preferred Reporting Items for Systematic Reviews and Meta-Analyses (PRISMA) guidelines, the search yielded 397 studies (159 from PubMed, 71 from Embase, 48 from Cochrane Library, and 119 from Google Scholar). After the removal of 133 duplicate records, 264 records were screened based on titles and abstracts. Following the review of titles and abstracts, 227 records were excluded because they did not meet our criteria. Upon further evaluation, 33 records were excluded for not meeting the eligibility criteria as follows: 11 due to incorrect study design, 16 for irrelevant interventions, and six for outcomes not of interest. This resulted in the inclusion of four studies in the final review, which were deemed relevant for detailed analysis. The summary of the study selection process is provided in Figure 1.

Postoperative Analgesic Consumption

Magnesium sulfate demonstrated varying efficacy in reducing postoperative analgesic consumption across the studies. Kumar et al. reported that total analgesic consumption over 24 hours was significantly lower in the dexmedetomidine group (142.50 ± 22.88 mg) compared to the magnesium group (197.50 ± 36.76 mg), with a highly significant difference (p < 0.001) [18]. Demiroglu et al. found that tramadol consumption was significantly lower in the intramuscular (IM) magnesium group compared to the control group at multiple postoperative time points (p < 0.05). Comparisons between the intravenous (IV) magnesium and control groups showed no significant differences, except at select time intervals where the IV group showed lower consumption than the control group (p < 0.05) [19]. Apan et al. observed a longer time for the first analgesic request in the magnesium group compared to the control group (p = 0.04) and significantly reduced total analgesic consumption (p = 0.02) [20]. In contrast, Ghaffaripour et al. found no statistically significant difference in total postoperative PCA morphine consumption between the magnesium and placebo groups (0.59 ± 0.04 mg/kg vs. 0.7 ± 0.08 mg/kg, p = 0.23) [21]. The characteristics of the studies that are part of this review are displayed in Table 2.

**Table 2:** Summary of the studies' characteristics. MgSO_4_: magnesium sulfate; IV: intravenous; IM: intramuscular

Studies	Study design	Sample size	Dose	Comparison	Key findings
Kumar et al. (2023) [18]	Prospective randomized double-blind	60 patients aged between 18 and 65 years	500 mg/kg magnesium sulfate equivalent to 1 mL	Dexmedetomidine	When ropivacaine and magnesium sulfate wound infiltration are used during the postoperative phase following lumbar laminectomy procedures, it may result in more effective analgesia, a reduction in the need for additional analgesics, and higher patient satisfaction
Demiroglu et al. (2016) [19]	Prospective randomized double-blind	75 patients aged between18 and 65 years	IV group = 50 mg/kg MgSO_4_ in 150 mL saline in 30 mins, IM group = 50 mg/kg MgSO_4_ in 30 mL saline was injected into the paraspinal muscles	Placebo	Magnesium delivered directly to the surgical site was more successful in producing postoperative analgesia than magnesium given systemically
Apan et al. (2004) [20]	Prospective randomized double-blind	50 adult patients, ASA I-II	5 mg/kg bolus of magnesium sulfate followed by a 500 mg/h infusion	Placebo	Magnesium sulfate infusion extended the duration of postoperative analgesia provided by meperidine without influencing sedation levels
Ghaffaripour et al. (2016) [21]	Prospective randomized double-blind	40 patients aged between 18 and 60 years	Magnesium sulfate (30 mg/kg) by a maintenance dose of 10 mg/kg/h	Placebo	Magnesium sulfate infusion for laminectomy did not reduce the patients' pain or the amount of opioids they needed in the first 24 h following the procedure

Pain Intensity

Results on pain intensity as measured by VAS or NRS scores varied among the studies. For instance, Kumar et al. observed that both groups' VAS scores were comparable until the 6 hours where the magnesium group scores (mean of 4.67 ± 0.48) were significantly higher than the dexmedetomidine group (mean of 1.77 ± 0.43, p < 0.001) [18]. Although having a higher VAS score at the 12-hour mark, the magnesium group displayed lower VAS ratings (mean of 2.53 ± 1.01) [16]. At all postoperative time points, Demiroglu et al. found no statistically significant changes in numeric pain rating scale (NRS) scores between the groups (p > 0.05) [19]. Apan et al. discovered that, for the first 24 hours, the VAS scores of the two groups were similar, except for the 12 hours, when the magnesium group's values were significantly lower (p = 0.03) [20]. Ghaffaripour et al. observed no significant differences in the patterns of VAS scores at 6, 12, 18, and 24 hours after surgery between the magnesium and control groups (p = 0.52) [21].

Adverse Events

Adverse events were reported inconsistently across the studies. According to Kumar et al., neither group experienced any negative side effects, including dry mouth, respiratory depression, allergies, nausea and vomiting, or urine retention [18]. As stated in the study by Demiroglu et al., the control group experienced a significantly higher incidence of nausea and vomiting (36%) than the magnesium-treated groups (p < 0.05) [19]. Apan et al. and Ghaffaripour et al. did not report any data on adverse events [20,21].

Risk of Bias

**Figure 2:**
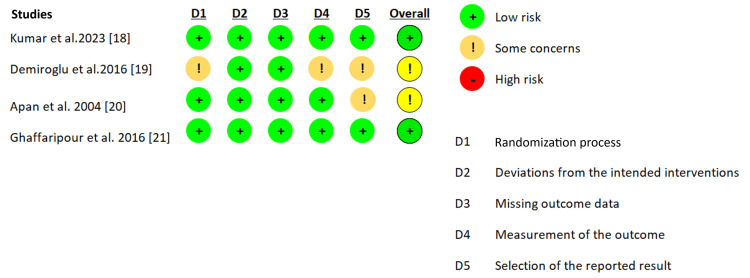
Risk of bias summary.

Figure 2 displays the summary for each article's risk of bias assessment using the revised Cochrane Collaboration tool (RoB 2) [18-21]. According to the authors' assessment, the two studies included were evaluated as having some concerns about the risk of bias. Due to the limited number of articles in each subgroup, the articles were included in the analysis regardless of their risk of bias score.

Discussion

Although magnesium sulfate is a well-known analgesic for its properties as an N-methyl-D-aspartate receptor blocker, there is sparse research on the analgesic effects of postoperatively administered IV magnesium sulfate [22]. The pre and perioperative administration of magnesium sulfate in spine surgeries has proven to be efficacious by mediating a great decrease in the use of opioids, length of stay, decreased visual analog scale scores, and improved overall patient satisfaction [23,24]. However, administration of the analgesic in the postoperative lumbar spine setting has yet to be thoroughly elucidated. This is the first systematic review covering the analgesic properties and pharmacological effects of magnesium sulfate on patients postlumbar spinal surgery. This systematic review's findings suggest that administration of magnesium sulfate in the postoperative setting has the potential to provide analgesic benefits, depending on the context of the lumbar spinal surgery at hand, however, the results may differ according to the method of administration and groups in comparison. According to Kumar et al., while magnesium sulfate remains advantageous in pain control, dexamethasone also provided significant analgesia as well as positive hemodynamic effects, which resulted in lesser heart rate and blood pressure postoperatively (p < 0.05) [18]. Of particular interest, in the dexamethasone group, the duration of analgesia lasted almost 2 hours longer after surgery (10.05 ± 1.62 hours) compared to the magnesium sulfate group (8.07 ± 1.83 hours), and the difference is both clinically and statistically significant (p < 0.05). However, the differences in pain intensity when measured by the visual analog score (VAS) showed variability between the two groups. For example, at 6 hours, the magnesium sulfate group achieved a lower VAS score (4.67 ± 0.48), but this benefit was not enduring at the 12-hour mark, while the VAS of the dexamethasone group was 5.00 ± 0.00, and enduring till the 12-hour mark [16].

Demiroglu et al.'s study revealed quite a different aspect for analysis concentrating on the method of magnesium sulfate administration. The intramuscular administration group demonstrated a significantly lower total tramadol consumption as compared to both intravenous administration and control groups (p < 0.05). Moreover, the results from the numeric pain rating scale (NRS) showed that the control group rated the pain as being more severe within the first 12 hours, which further supported the analgesic efficacy of magnesium sulfate administration in the postoperative setting, particularly utilizing the intramuscular route [17].

Apan et al.’s study revealed further support for the analgesic properties of magnesium sulfate in lumbar spine surgery patients, observing that magnesium sulfate patients witnessed a longer time to first analgesic request, as well as a statistically significant reduction in total analgesic consumption in comparison with control groups. Furthermore, VAS scores between control groups and magnesium sulfate groups varied significantly in the first 12 hours postoperatively, with a large reduction in reported pain compared with control groups at the same time, however, no marked difference was noted at the 24-hour mark. In terms of adverse events, no adverse events were recorded in the study groups, which limits this systematic review's ability to analyze the adverse effects of magnesium sulfate comprehensively [20].

Ghaffaripour et al. in contrast to the previous studies, found no statistically significant reduction in total postoperative morphine consumption in magnesium groups in comparison with placebo groups. Furthermore, no significant difference was noted in VAS scores across all time marks between the magnesium and control groups (p = 0.52). Similar to Apan et al.'s study, adverse effects were not reported, and therefore, this further limits the study's ability to report the adverse effects of the analgesic agents. Ghaffaripour et al. overall reported no clinically significant difference in the use of magnesium sulfate postoperatively as opposed to placebo groups among the same patients group [21].

The differing outcomes between these four studies highlight the complexity of magnesium sulfate's postoperative effects. Kumar et al. emphasized the comparative effectiveness of dexamethasone, as opposed to magnesium sulfate, while still highlighting the efficacy of both agents [18]. Demiroglu et al. illustrated that magnesium sulfate, particularly when administered intramuscularly, could largely reduce the need for supplementary analgesics, such as opioids [19]. Ghaffaripour et al., in contrast, reported that magnesium sulfate had no clinically significant effect on either pain or analgesic consumption and concluded that magnesium sulfate had no significant benefit in the lumbar spine surgery patients' demographic when administered postoperatively [21]. Apan et al. showed a significant reduction in VAS scores at the 12-hour mark, but not at the 24-hour mark, and a prolongation in time to the first analgesic request, demonstrating the efficacy of the agent [20]. This could be valuable in the avoidance of opioid-related side effects, by allowing lower doses of opioids to be administered. The results from these studies highlight the remarkable analgesic properties of magnesium sulfate postoperatively, while also shining light on the importance of optimizing administration routes and timing. Although significant findings have been illustrated in these studies, further research is required to comprehensively showcase the role and efficacy of postoperative magnesium sulfate administration in the lumbar spine surgery setting.

Study limitations

This systematic review, however, is not free of limitations. This study was confronted with a number of limitations, the primary limitation being the small number of studies eligible for inclusion in this systematic review, due to the scarcity of research in the area. Moreover, this study is limited by the heterogeneity of the studies included, in terms of analgesic route of administration, outcome reporting, and opposing control groups. Furthermore, the four studies that fit the eligibility criteria were marked with differences in methodology, routes, and forms of administration, patient demographics were slightly variable as well. Additionally, the small sample sizes in the studies may also act as a limitation, which may decrease the statistical power of the study. Furthermore, due to the difference in follow-up periods between the study groups, the ability to draw a definitive conclusion on long-term outcomes of postoperative analgesia on pain remedy might be limited. Finally, this review may be limited by confounding variables such as patient demographics, medications, and comorbid diseases in patients from either sample group.

## Conclusions

While magnesium sulfate does demonstrate potential as an analgesic additive in lumbar surgery patients to facilitate a reduction in postoperative pain and opioid consumption, a comprehensive assessment of the agent's efficacy has proven difficult due to the influence of external variables on the analysis of pain, analgesic consumption, and adverse events. Standardized research efforts are essential to define its optimal clinical applications and address existing knowledge gaps.

## References

[1] Abou-Setta AM, Beaupre LA, Rashiq S (2011). Comparative effectiveness of pain management interventions for hip fracture: a systematic review. Ann Intern Med.

[2] Kehlet H (1994). Postoperative pain relief - what is the issue?. Br J Anaesth.

[3] Kara H, Sahin N, Ulusan V, Aydogdu T (2002). Magnesium infusion reduces perioperative pain. Eur J Anaesthesiol.

[4] Albrecht E, Kirkham KR, Liu SS, Brull R (2013). Peri-operative intravenous administration of magnesium sulphate and postoperative pain: a meta-analysis. Anaesthesia.

[5] Arumugam S, Lau CS, Chamberlain RS (2016). Perioperative adjunct magnesium decreases postoperative opioid requirements - a meta-analysis. Int J Clin Med.

[6] McCarthy RJ, Kroin JS, Tuman KJ, Penn RD, Ivankovich AD (1998). Antinociceptive potentiation and attenuation of tolerance by intrathecal co-infusion of magnesium sulfate and morphine in rats. Anesth Analg.

[7] Srebro D, Vuckovic S, Milovanovic A, Kosutic J, Vujovic KS, Prostran M (2017). Magnesium in pain research: state of the art. Curr Med Chem.

[8] Elsharnouby NM, Eid HE, Abou Elezz NF, Moharram AN (2008). Intraarticular injection of magnesium sulphate and/or bupivacaine for postoperative analgesia after arthroscopic knee surgery. Anesth Analg.

[9] Dabbagh A, Elyasi H, Razavi SS, Fathi M, Rajaei S (2009). Intravenous magnesium sulfate for post-operative pain in patients undergoing lower limb orthopedic surgery. Acta Anaesthesiol Scand.

[10] (2018). Spine surgery: global trends & opportunities. Spine Surgery | Global Trends & Opportunities.

[11] Bajwa SJ, Haldar R (2015). Pain management following spinal surgeries: an appraisal of the available options. J Craniovertebr Junction Spine.

[12] Bianconi M, Ferraro L, Ricci R (2004). The pharmacokinetics and efficacy of ropivacaine continuous wound instillation after spine fusion surgery. Anesth Analg.

[13] Bhaskar SB, Bajwa SJ (2013). Pharmaco-genomics and anaesthesia: mysteries, correlations and facts. Indian J Anaesth.

[14] Loftus RW, Yeager MP, Clark JA, Brown JR, Abdu WA, Sengupta DK, Beach ML (2010). Intraoperative ketamine reduces perioperative opiate consumption in opiate-dependent patients with chronic back pain undergoing back surgery. Anesthesiology.

[15] Moher D, Shamseer L, Clarke M (2015). Preferred Reporting Items for Systematic Review and Meta-Analysis Protocols (PRISMA-P) 2015 statement. Syst Rev.

[16] Sterne JA, Savović J, Page MJ (2019). RoB 2: a revised tool for assessing risk of bias in randomised trials. BMJ.

[17] Piggott T, Morgan RL, Cuello-Garcia CA, Santesso N, Mustafa RA, Meerpohl JJ, Schünemann HJ (2020). Grading of Recommendations Assessment, Development, and Evaluations (GRADE) notes: extremely serious, GRADE's terminology for rating down by three levels. J Clin Epidemiol.

[18] Kumar M, Singh RB, Vikal JP, Yadav JB, Singh D (2023). Comparison of ropivacaine plus dexmedetomidine and ropivacaine plus magnesium sulfate infiltration for postoperative analgesia in patients undergoing lumbar spine surgeries. Cureus.

[19] Demiroglu M, Ün C, Ornek DH (2016). The effect of systemic and regional use of magnesium sulfate on postoperative tramadol consumption in lumbar disc surgery. Biomed Res Int.

[20] Apan A, Buyukkocak U, Ozcan S, Sari E, Basar H (2004). Postoperative magnesium sulphate infusion reduces analgesic requirements in spinal anaesthesia. Eur J Anaesthesiol.

[21] Ghaffaripour S, Mahmoudi H, Eghbal H, Rahimi A (2016). The effect of intravenous magnesium sulfate on post-operative analgesia during laminectomy. Cureus.

[22] Soleimanpour H, Imani F, Dolati S, Soleimanpour M, Shahsavarinia K (2022). Management of pain using magnesium sulphate: a narrative review. Postgrad Med.

[23] Dehkordy ME, Tavanaei R, Younesi E, Khorasanizade S, Farsani HA, Oraee-Yazdani S (2020). Effects of perioperative magnesium sulfate infusion on intraoperative blood loss and postoperative analgesia in patients undergoing posterior lumbar spinal fusion surgery: a randomized controlled trial. Clin Neurol Neurosurg.

[24] Salkaya A, Oba S, Altınay M, Türk HŞ, Kılınç L, Yılmaz A (2024). The effects of perioperative low-dose magnesium sulfate infusion on postoperative pain in lumbar surgery. Signa Vitae.

